# Enhancing the resistant starch content of cassava starch via heat-moisture treatment for application as a prebiotic in chicken feed

**DOI:** 10.1016/j.vas.2026.100630

**Published:** 2026-03-23

**Authors:** Watcharamon Prasert, Nattaphong Akrimajirachoote, Montri Pattarapanawan, Attawit Kovitvadhi, Sukanya Phuengjayaem, Haibo Qin, Bing-Zheng Li, Nitnipa Soontorngun, Niyada Lansubsakul, Sathita Areerat, Ditpon Kotatha

**Affiliations:** a Division of Biochemical Technology, School of Bioresources and Technology, King Mongkut’s University of Technology Thonburi (Bangkhunthian Campus), Bangkok, 10150, Thailand; b Department of Physiology, Faculty of Veterinary Medicine, Kasetsart University, Bangkok, 10900, Thailand; c Department of Anatomy, Faculty of Veterinary Medicine, Kasetsart University, Bangkok, 10900, Thailand; d KU Vet Innova Nutricare Co., Ltd., Faculty of Veterinary Medicine, Kasetsart University, Bangkok, 10900, Thailand; e Department of Microbiology, Faculty of Science, King Mongkut’s University of Technology Thonburi, Bangkok, 10140, Thailand; f Institute of Grand Health, Guangxi Academy of Sciences, Nanning, 530007, China

**Keywords:** Heat-moisture treatment, Cassava starch, Resistant starch, Prebiotic, Broiler, Microbiome

## Abstract

This study aimed to increase the resistant starch (RS) content of cassava starch via heat-moisture treatment (HMT) and evaluate its prebiotic potential in chicken feed. The HMT involved autoclaving cassava starch at 20% moisture content and 121°C for 1 h (HMT-20), which yielded a high RS content (20.7%) in the cooked starch. In vitro fermentation with *Limosilactobacillus reuteri* TBRC291 demonstrated the promising prebiotic potential of cooked HMT-20, comparable to that of commercial fructo-oligosaccharides, as indicated by enhanced bacterial growth, reduced pH, and increased short-chain fatty acid (SCFA) production. A 1% concentration of cooked HMT-20 was identified as optimal for prebiotic use. In the in vivo assessment, chickens were given feed supplemented with 1% cooked HMT-20 (HMT-Feed), displaying no adverse effects on growth performance over 35 days and significantly reducing relative abdominal fat and heart weight while increasing breast weight. Meat from the HMT-Feed group also exhibited increased yellowness and reduced cooking loss. Histological analysis revealed an improved intestinal morphology, including a greater villus height, reduced crypt depth, and higher villus-to-crypt ratio. Additionally, the HMT-Feed group exhibited a lower cecal pH and higher SCFA levels (notably butyric acid). Deep gut microbiota profiling revealed enriched levels of lactic acid- and butyrate-producing bacteria, indicating improved gut health. The HMT-modified product demonstrates potential applicability as a value-added and sustainable feed additive in the poultry industry.

## 1 Introduction

Cassava, or tapioca (*Manihot esculenta* Crantz), is a major root crop widely used in the food, feed, and industrial sectors and represents an economically important raw material for starch production, particularly in Thailand, where annual cassava starch exports reach approximately 2 million tons (Sriroth et al., 2000). However, the inherent molecular structure of native cassava starch limits its functional properties and direct industrial use (Zhu, 2015). To address these limitations, chemical, physical, and enzymatic modification techniques have been applied to enhance starch functionality, enabling the development of value-added products such as sour starch and resistant starch for food and livestock feed applications (Parmar et al., 2017; Zhu, 2015).

Physical modification offers a cost-effective, safe, and environmentally friendly alternative to chemical and enzymatic processes, making it particularly suitable for animal feed applications where regulatory acceptance, feed safety, and production cost are critical. Among these methods, heat–moisture treatment (HMT) is a prominent hydrothermal technique that modifies starch functionality while preserving its molecular integrity (Fonseca et al., 2021). HMT involves heating starch at relatively high temperatures (80–140°C) under low moisture conditions (<35% w/w) for a specified period (Li et al., 2020). This process induces structural changes, including the disruption and reassociation of crystalline regions, extension of double helices, and strengthening of interactions between amylose and amylopectin chains (Fonseca et al., 2021).

A key outcome of HMT is the promotion of resistant starch (RS) formation through starch structural reorganization, thereby reducing susceptibility to enzymatic digestion. It is not hydrolyzed to D-glucose in the small intestine within 120 min of consumption, thereby reaching the colon intact (Fuentes‐Zaragoza et al., 2011). As a nondigestible carbohydrate, RS is fermented by intestinal microorganisms, resulting in the production of short-chain fatty acids (SCFAs), mainly acetate, propionate, and butyrate, among which butyrate serves as an important energy source for colonocytes and contributes to colonic health (Kotatha et al., 2023).

In poultry production systems, antibiotic growth promoters (AGPs) have historically been used to enhance growth performance and maintain intestinal health. However, increasing regulatory restrictions and concerns over antimicrobial resistance have led to their global withdrawal, prompting the search for alternative nutritional strategies (Islam et al., 2022; Sultan et al., 2024; Zada et al., 2024). Consequently, gut-modulating dietary components such as RS have gained increasing attention. Indeed dietary RS has been associated with alterations in cecal microbial composition, particularly reductions in Firmicutes, accompanied by decreased hepatic fatty acid synthesis and reduced abdominal fat deposition during early growth (Zhang et al., 2020b). RS supplementation has also been reported to influence gut health and growth performance through modulation of cecal SCFA production and nutrient digestion (Oluseyifunmi et al., 2024), and higher dietary levels of RS have been linked to enhanced hepatic lipolysis and gluconeogenesis, contributing to increased whole-body energy expenditure during the grower phase (Liu et al., 2020b). Collectively, these findings support the consideration of RS as a prebiotic substrate in poultry nutrition.

According to the International Scientific Association for Probiotics and Prebiotics (ISAPP), prebiotics are defined as substrates that are selectively utilized by host microorganisms, conferring a health benefit (Gibson et al., 2017). As nondigestible compounds, prebiotics escape digestion and absorption in the upper gastrointestinal tract and reach the colon intact. The colon serves as the principal site of prebiotic activity, where these compounds are selectively fermented by beneficial microorganisms, particularly *Bifidobacteria* and *Lactobacilli*, leading to enhanced microbial proliferation and metabolic activity and the production of SCFAs (Alshelmani et al., 2021; 2016a; 2016b). While this definition emphasizes selective utilization, the degree to which such selectivity is demonstrated may vary depending on the experimental model and microbiological resolution in in vivo studies.

To the best of our knowledge, limited information is available regarding the experimental evaluation of HMT cassava starch as a potential prebiotic for chicken feed. Accordingly, this study aimed to enhance the RS content of cassava starch via HMT and evaluate its prebiotic efficacy. Further, the HMT-modified product was assessed using both in vitro fermentation and in vivo animal trials. The in vivo investigation included evaluations of growth performance, carcass traits, meat quality, and gastrointestinal tract characteristics, along with an in-depth analysis of gut microbiota composition.

## 2 Materials and methods

### 2.1 Materials

Cassava starch was supplied by Banpong Tapioca Flour Industrial Co., Ltd. (Ratchaburi, Thailand). A single production batch of cassava starch was used throughout all experimental stages, including starch modification, in vitro fermentation, and in vivo animal trials. α-Amylase powder (type VI-B from the porcine pancreas, 13 U/mg) and amyloglucosidase (EC 3.2.1.3 from *Aspergillus niger*, 300 U/mL) were obtained from Sigma-Aldrich (Missouri, USA); glucose liquicolor was acquired from Stanbio Laboratory (Boerne, USA); and a total starch assay kit was purchased from Megazyme (Bray, Ireland). Commercial fructo-oligosaccharides (FOS) were sourced from a local company in Bangkok (Thailand), and *Limosilactobacillus reuteri* TBRC291 was purchased from the National Center for Genetic Engineering and Biotechnology (Pathum Thani, Thailand). One-day-old broiler chicks of the Avian CP breed were provided by Charoen Pokphand Foods PCL (Bangkok, Thailand). An MGIEasy Magnetic Beads Genomic DNA Extraction Kit was purchased from MGI Tech Co., Ltd. (Shenzhen, China). The ingredients used for the chicken feed were procured from a local supplier in Thailand, and the vitamin and mineral premix formulations were procured from Feed Specialties Co., Ltd. (Pathumthani, Thailand). The chemicals and solvents used in this work were of analytical grade.

### 2.2 HMT modification

Cassava starch was modified via HMT following the method of Li et al. (2020), with a few modifications. A total of 100 g of cassava starch was soaked in 300 mL of water and incubated at 4°C for 24 h. Excess water from the equilibrated slurry was removed by filtration using a No. 4 glass filter under vacuum suction. After filtration, the starch cake was sieved using a 2-mm sieve, and its moisture content was adjusted by air-drying to 15, 20, 25, and 30% (±2%), as verified using a moisture analyzer (Sartorius, model MA35, Germany). The samples were then placed in polyethylene bags, sealed, and autoclaved at 121°C for 1 h. After cooling to room temperature, the samples were sieved again and dried in an oven at 40°C for 24 h. The HMT-modified products were designated based on their moisture content as HMT-15, HMT-20, HMT-25, and HMT-30, corresponding to samples modified at moisture levels of 15, 20, 25, and 30%, respectively.

### 2.3 RS content analysis

The RS content of the uncooked and cooked samples was determined using the method of Englyst et al. (1992), with some modifications. The starch fractions were categorized as follows: rapidly digestible starch (RDS), which is hydrolyzed into glucose within 20 min; slowly digestible starch (SDS), which is completely digested into glucose between 20 and 120 min; and RS, which is resistant to enzymatic digestion within 120 min.

First, 100 mg of an uncooked sample, 10 glass beads (0.5 mm in diameter), and 8 mL of a sodium acetate buffer (0.1 M, 4 mM CaCl_2_, pH 5.2) were added to an Erlenmeyer flask, and the mixture was incubated at 37°C for 10 min to ensure equilibration. Subsequently, 2 mL of an enzyme mixture containing α-amylase (900 U) and amyloglucosidase (7.5 U) in the sodium acetate buffer was added. The flask was then incubated at 37°C with agitation at 130 strokes/min. After 20 and 120 min, a 1-mL aliquot was immediately boiled at 100°C for 10 min to inactivate the enzymes. The solution was then centrifuged at 2000 × *g* for 5 min, and the liberated glucose in the supernatant was quantified. The glucose released at 20 min (G20) and 120 min (G120) was determined by reacting a 10-µL aliquot with 1 mL of glucose liquicolor at 37°C for 5 min, followed by the measurement of absorbance at 500 nm using a spectrophotometer (Thermo Fisher Scientific, model Genesys 20, USA). Additionally, the total starch (TS) content was determined using the AOAC Official Method 996.11 with a total starch assay kit (AOAC International, 2005). The RS content was calculated using Eqs. (1)–(3) (Sari et al., 2022):

(1) RDS = G20 × 0.9

(2) SDS = (G120 - G20) × 0.9

(3) RS = TS - (RDS + SDS)

To determine the RS in the cooked sample, 100 mg of the sample, 10 glass beads, and 2 mL of distilled water were added to an Erlenmeyer flask and boiled at 100°C for 10 min. Subsequently, 6 mL of a sodium acetate buffer (0.1 M, 4 mM CaCl_2_, pH 5.2) was added, and the mixture was then equilibrated by incubation at 37°C for 10 min. The procedure was then continued as described in the preceding steps.

### 2.4 Evaluation of prebiotic potential

The prebiotic potential of the HMT-modified product was evaluated and compared to that of commercial prebiotic FOS via in vitro fermentation, following the method of Hayati et al. (2025) and Pattarapanawan et al. (2025). The growth dynamics of the probiotic, pH changes, and SCFA production at 24 h of fermentation were examined. Prior to in vitro fermentation, all samples were cooked and subjected to in vitro enzymatic digestion following the procedure for RS content analysis, with modifications to scale up the process for a 100 g sample. After incubation for 120 min, the undigested residue was collected, washed twice with distilled water, and dried.

In this study, *L. reuteri*, the most abundant *Lactobacillus* species in the chicken gastrointestinal tract (Wang et al., 2014), was selected as the representative probiotic. *L. reuteri* was cultured in selective de Man, Rogosa, and Sharpe (MRS) broth at 37°C for 24 h, and the initial bacterial cultures were subsequently adjusted to a concentration of 1 × 10^8^ CFU/mL for use as test cultures. A 1% (v/v) inoculum was introduced into the MRS broth supplemented with various carbon sources. The carbon source was also substituted with 2% HMT-modified product, 2% FOS, and 2% native starch (undigested, uncooked) for comparison. Prior to inoculation, the carbon sources were sterilized for 15 min using ultraviolet radiation (Hongkulsup et al., 2025). The MRS broth containing 2% glucose served as the control. The cultures were incubated at 37°C, and viable cell counts were determined at 0, 24, and 48 h using the drop-plate method on MRS agar. The pH of the culture media was measured using a pH meter (Hanna, model HI98103, USA); additionally, a 1 mL aliquot of the culture media was immediately placed in a freezer at -20°C to halt fermentation for the SCFA analysis.

The SCFAs, including acetic, propionic, and butyric acids, were analyzed using high-performance liquid chromatography (HPLC; Shimadzu, model UFLC, Japan), following the method described by Wandee et al. (2015). The frozen fermentation broth was rapidly thawed in warm water and filtered through a 0.45 µm nylon membrane. Subsequently, 20 µL of the filtered solution was injected into the HPLC system, consisting of a liquid chromatograph (LC-20AD); a refractive index detector (RID-10A); and a column oven (CTO-20A), equipped with a VertiSep OA 8 µm 7.8 × 300 mm column (Vertical Chromatography, Thailand). The sample was analyzed in isocratic mode using 0.005 N sulfuric acid as the mobile phase at a flow rate of 0.6 mL/min, and the column temperature was maintained at 60°C. The data analysis was carried out using LabSolutions software. Acetic, propionic, and butyric acids were used as external standards.

### 2.5 Determination of optimal concentration of HMT-modified products in chicken feed

The optimal concentration of the HMT-modified product in the chicken feed was evaluated via in vitro fermentation, using a method similar to that described for evaluating the prebiotic potential (Section 2.4). Various concentrations of the HMT-modified product (0.5, 1, 2, 3, and 4%) were used as the carbon source.

### 2.6 Animal experimentation

#### 2.6.1 Animal ethics

All animal experiments were conducted strictly according to the guidelines of animal care and use under the Ethical Review Board of the Office of the National Research Council of Thailand (NRCT) and approved by the Institutional Animal Care and Use Committee of King Mongkut’s University of Technology Thonburi (KMUTT-IACUC-2024-011) and Kasetsart University (ACKU67-VET-048).

#### 2.6.2 Animal diet and husbandry

The animal husbandry protocol was performed following the guidelines of Kongsup et al. (2022). A total of 168 1-day-old healthy male broiler chicks (Avian CP) were randomly assigned to two experimental groups following a completely randomized design (CRD), with seven replicates per group and 12 birds per replicate. The control group (CON) received feed without the HMT-modified product, while the treatment group (HMT-Feed) was given feed containing the HMT-modified product.

The broilers were reared in a closed system at the Laboratory Animal Center, Faculty of Veterinary Medicine, Kasetsart University. All broilers were housed in floor cages (1 × 1 m enclosures) with rice hull bedding, maintaining a humidity- and ventilation-controlled environment. A regulated lighting program (12 h light: 12 h dark) was also implemented. The ambient temperature was maintained at 34°C for the first 5 days and then gradually reduced by 2°C per week to reach 25°C; this was maintained until the conclusion of the study on day 35. All broilers were provided *ad libitum* access to feed and clean water. The corn and soybean meal-based diet for the broilers was formulated in two phases: starter (days 1–21) and finisher (days 22–35). All diets within each phase were formulated to be isocaloric and isonitrogenous and to meet the nutrient requirements of broiler chickens during the starter and finisher phases, as outlined in the ROSS® guidelines (Aviagen, 2022). All feed ingredients were thoroughly mixed and the diets were prepared in mash form without any additional heat processing. The only exception was the HMT-modified product, which was pre-cooked prior to its inclusion in the diet, with this treatment also incidentally reflecting the thermal conditioning commonly applied in commercial pelleted feed production. The ingredients and chemical compositions of the experimental diets are presented in Table 1. The metabolizable energy was calculated following the method of Tay-Zar et al. (2024), while the chemical compositions, including the crude protein, crude fat, crude fiber, and ash contents, were determined using the methods of the Association of Official Analytical Chemists (AOAC International, 2005).

**Table 1:** Ingredients, chemical composition, and RS content of experimental diets.

Item		Starter diet (days 1–21)		Finisher diet (days 22–35)	
		CON	HMT-Feed	CON	HMT-Feed
Ingredients (%)					
	Corn	45.8	45.8	57.8	57.8
	Dehulled soybean meal	39.3	39.3	28.0	28.0
	Palm oil	6.06	6.06	5.73	5.73
	Cooked native cassava starch	5.00	-	5.00	-
	Cooked HMT-modified product	-	5.00	-	5.00
	Dicalcium phosphate	1.38	1.38	0.91	0.91
	Limestone	1.38	1.38	1.27	1.27
	Salt	0.24	0.24	0.19	0.19
	Vitamin premix^1^	0.16	0.16	0.16	0.16
	Mineral premix^2^	0.20	0.20	0.20	0.20
	L-Lysine HCl	-	-	0.17	0.17
	DL-Methionine	0.21	0.21	0.30	0.30
	L-Threonine	-	-	0.04	0.04
	Sodium bicarbonate	0.20	0.20	0.20	0.20
	Choline chloride	0.07	0.07	0.07	0.07
Analyzed composition (%DM)					
	Crude protein	25.2	25.3	21.2	21.6
	Ether extract	7.49	7.32	9.49	9.19
	Crude ash	6.42	5.82	5.15	4.91
	Crude fiber	2.66	2.90	2.36	2.33
Calculated composition (%DM)					
	RS	0.62	1.04	0.62	1.04
	Crude protein	25.0	25.0	21.0	21.0
	Metabolizable energy (kcal/kg)	3100	3100	3230	3230
	Methionine	0.58	0.58	0.53	0.53
	Methionine+Cysteine	0.94	0.91	0.84	0.80
	Available phosphorus	0.45	0.45	0.38	0.38

HMT= heat-moisture treatment; CON= control group, received feed without the HMT-modified product; HMT-Feed= treatment group, given feed containing the HMT-modified product; DM= dry matter; RS= resistant starch.

1 Vitamin premix was supplied per kilogram of premix at 2500,000 IU of vitamin A; 1000,000 IU of vitamin D3; 7000 IU of vitamin E; 700 mg of vitamin K; 400 mg of vitamin B1; 800 mg of vitamin B2; 400 mg of vitamin B6; 4 mg of vitamin B12; 30 mg of biotin; 3111 mg of Ca pantothenate; 100 mg of folic acid; 15,000 mg of vitamin C; and 5600 mg of vitamin B3.

2 Mineral premix was supplied per kilogram of premix at 10,500 mg of Zn; 10,920 mg of Fe; 9960 mg of Mn; 3850 mg of Cu; 137 mg of I; and 70 mg of Se.

#### 2.6.3 Growth performance

Body weight (BW) and feed intake (FI) were measured on days 1, 14, 21, 28, and 35 to calculate average daily gain (ADG), average daily feed intake (ADFI), and feed conversion ratio (FCR). Morbidity and mortality were recorded daily. BW and FI were recorded on a pen basis and expressed as averages per bird within each pen. ADG, ADFI, and FCR were subsequently calculated per bird within each pen, with the pen considered the experimental unit for statistical analysis, as described in Eqs. (4)–(6):

(4) $$\text{ADG}\;=\;\frac{\text{Total}\;\text{BW}\;\text{gain}\;\text{in}\;\text{the}\;\text{pen}\;(g)}{\text{Number}\;\text{of}\;\text{days}\;\text{fed}\;(\text{days})\;\times \;\text{Number}\;\text{of}\;\text{chickens}\;\text{per}\;\text{pen}}$$

(5) $$\text{ADFI}\;=\;\frac{\text{Total}\;\text{feed}\;\text{intake}\;\text{in}\;\text{the}\;\text{pen}\;(g)}{\text{Number}\;\text{of}\;\text{days}\;\text{fed}\;(\text{days})\;\times \;\text{Number}\;\text{of}\;\text{chickens}\;\text{per}\;\text{pen}}$$

(6) $$\text{FCR}\;=\;\frac{\text{ADFI}\;}{\text{ADG}}$$

#### 2.6.4 Slaughter and sample collection

On day 35, all broilers were euthanized using CO_2_ and immediate subsequent exsanguination of the unconscious broilers (Underwood & Anthony, 2020). Thereafter, the broilers were immersed in hot water at 60°C for 5 s to facilitate feather removal. The feet, head, spleen, and gastrointestinal tract were then excised to evaluate the carcass traits and meat quality. The gastrointestinal tract of two broilers with body weights closest to the average body weight of the pen was selected from each replicate (Rahmatnejad & Saki, 2016). Cecal contents were collected from these broilers for pH measurements, and a separate portion was immediately collected and stored at -20°C for the cecal fermentation-derived SCFA and cecal microbiota analyses. Additionally, the duodenum, jejunum, and ileum were collected for gastrointestinal tract characterization and histological structure analysis.

#### 2.6.5 Carcass traits

The slaughter weight was measured immediately after slaughtering the broilers. The hot carcass weight (HCW) was recorded following the removal of the head, feet, spleen, abdominal fat, and gastrointestinal tract; the weights of these body parts were subsequently measured and expressed as a percentage of the HCW. The carcasses were then chilled at 4°C for 24 h, after which the cold carcass weight (CCW) was recorded. The heart, liver, wings, breast, and thighs were dissected and weighed, expressing their weights as a percentage of the CCW. Additionally, the meat-to-bone ratio was determined using the thigh samples. The CCW was divided by the slaughter weight and multiplied by 100 to calculate the dressing-out percentage.

#### 2.6.6 Characterization of meat quality

The meat quality was assessed using breast samples; their pH was measured 45 min after slaughter (pH_45_) using a meat pH meter (Hanna, model HI99163, USA), and the samples were then stored at 4°C. After 24 h, the samples were equilibrated to room temperature for 30 min before measuring the ultimate pH (pH_u_). Subsequently, the surface color of the samples was measured using a colorimeter (FRU, model WF32, China) with the following settings: a D65 illuminant, a 10° standard observer angle, and an 8 mm aperture. The color parameters were recorded using the CIELAB system: L* (lightness), a* (redness), and b* (yellowness). The mean of three random measurements taken at different points within each sample was used to represent the pH and color values. Thereafter, the breast samples were cut into 5 × 5 × 2 cm segments to determine the water-holding capacity (WHC), which was assessed sequentially by measuring the drip loss, thawing loss, and cooking loss according to the method of Kotatha et al. (2025).

#### 2.6.7 Gastrointestinal tract characterization and histological structure analysis

Three segments of the small intestine (duodenum, jejunum, and ileum) were collected to evaluate the segment length, villus height, crypt depth, and villus-to-crypt ratio. The relative length of each segment was calculated based on the ratio of the intestinal segment length to the BW. Next, 2 cm samples were obtained from the mid-regions of each intestinal segment, longitudinally incised, and rinsed with normal saline to remove the luminal contents. The tissues were then affixed to cardboard through the serosal surface to maintain the proper orientation and gently distend the tissues. The samples were fixed in 10 % neutral buffered formalin for 24 h, followed by standard paraffin embedding. The tissue blocks were sectioned at a thickness of 4 μm and stained with hematoxylin and eosin (H&E) (Pattarapanawan et al., 2020). The histological slides were examined using an inverted microscope (Nikon, model ECLIPSE, Japan). The images were captured and analyzed using NIS-Elements D Imaging Software (version 5.42.06, Build 182 LO, 64-bit; Nikon, Japan). At least 20 well-oriented villi and their corresponding crypts were measured for each intestinal section.

#### 2.6.8 Cecal pH and cecal fermentation-derived SCFAs

The cecal pH was measured using a pH meter (Hanna, model HI981036, USA) at room temperature. Cecal samples for the determination of fermentation-derived SCFAs were prepared according to the guidelines of Walugembe et al. (2015), with some modifications. The frozen cecal samples were rapidly thawed in warm water, and approximately 1.0 g of the cecal content was transferred to a 10 mL tube. Thereafter, 4 mL of 24% meta-phosphoric acid in 0.005 N sulfuric acid was added, and the mixture was homogenized using a vortex mixer. The sample was then centrifuged at 11,000 × *g* for 15 min at 4°C. The supernatant was filtered through a 0.45 µm nylon membrane, and 20 µL was injected into an HPLC system. The same protocol described in Section 2.4 was followed.

#### 2.6.9 Cecal microbiota analysis

Fecal DNA was extracted using the MGIEasy Magnetic Beads Genomic DNA Extraction kit following the manufacturer’s instructions. The full-length 16S rRNA gene (∼1500 bp) was amplified using ONT adapter-tailed universal primers targeting the 27F/1492R regions: 16S-F (5′-GTCTCGTGGGCTCGGAGAGTTTGATCCTGGCTCAG-3′) and 16S-R (5′-TCGTCGGCAGCGTCGGTTACCTTGTTACGACTTC-3′). Amplicons were sequenced on the Oxford Nanopore Technologies PromethION platform using R10.4.1 flow cells, targeting ≥50,000 reads per sample.

Basecalling and demultiplexing were performed using Dorado. Primers and adapters were trimmed with pychopper (v2), and reads were quality-filtered using chopper, removing sequences with a median Q-score <10 or lengths outside 1200–1800 bp. Clean reads were classified to the species level using emu, and abundance tables were generated using a custom Python script.

Alpha diversity indices (Chao1, Shannon–Weaver, and Simpson) and beta diversity (Bray–Curtis distance with PCA visualization) were calculated using the vegan package (v2.5.6). Beta diversity differences were tested using PERMANOVA (adonis function). For taxonomic comparisons, relative abundances at the phylum level, Firmicutes/Bacteroidetes ratio, total Lactobacillus spp., the top 20 most abundant species, and taxa with relative abundance >0.01% were analyzed between groups using Student’s *t*-test after centered log-ratio (CLR) transformation (Rcmdr v2.9–5). Differentially abundant taxa were further identified using LEfSe (v1.18.0).

### 2.7 Statistical analysis

For each HMT condition, three independently prepared samples were used (*n* = 3), and the RS content of each sample was measured in triplicate. In vitro fermentation experiments were conducted using three independent culture tubes per treatment (*n* = 3), with growth profiles, pH changes, and SCFA production measured in triplicate for each tube. For all analyses, the mean value of the technical replicates was considered as one independent replicate. Results are expressed as mean ± standard deviation (SD). Statistical analysis was performed using one-way ANOVA followed by Tukey’s post-hoc test, with significance set at *p* < 0.05.

In the animal experiments, the results were presented as the mean and pooled standard error of the mean (SEM). All continuous data on the growth performances, carcass traits, and other aspects were analyzed with the Student’s *t*-test, with the groups serving as the fixed factor. If the dependent variables were not accepted on the homogeneity of variance from Levene’s test, the Welch's *t*-test was used instead. Statistically significant differences were accepted at *p* < 0.05. The statistical analyses were primarily conducted using SPSS software (version 18; SPSS Inc., IL, USA), while the analyses of fecal microbiota were performed using R (RStudio IDE, version 2025.05.1 + 513).

## 3 Results and discussion

### 3.1 RS content analysis

The RS content of the native and HMT-modified products in both the uncooked and cooked samples is presented in Table 2. In the uncooked samples, native starch exhibits the highest RS content (66.2%), while the RS content in the uncooked HMT-modified products decreases with an increasing moisture content, from 62.6% (HMT-15) to 19.6% (HMT-30). After cooking, the RS content of native starch markedly decreases, reaching 12.4%. Among the HMT-modified products, HMT-20 retains the highest RS content after cooking (20.7%), whereas lower values were observed in HMT-15 (13.8%), HMT-25 (15.2%), and HMT-30 (14.2%).

**Table 2:** RS content of native starch and HMT-modified products in uncooked and cooked samples.

Sample	RS content		
	Uncooked		Cooked
Native starch	66.2 ± 0.6^a^		12.4 ± 1.5^b^
HMT-15	62.6 ± 1.2^b^		13.8 ± 3.7^b^
HMT-20	44.4 ± 2.1^c^		20.7 ± 0.8^a^
HMT-25	24.2 ± 2.6^d^		15.2 ± 5.5^b^
HMT-30	19.6 ± 1.9^e^		14.2 ± 1.5^b^

RS = resistant starch; HMT = heat-moisture treatment. The numbers 15, 20, 25, and 30 in the sample names represent the moisture content (%). The data were analyzed using a one-way ANOVA, and statistically significant differences among the treatment groups were determined using Duncan’s post hoc test. Different lowercase letters (a, b, …) within the same column indicate significant differences (*p* < 0.05).

**Fig. 1:**
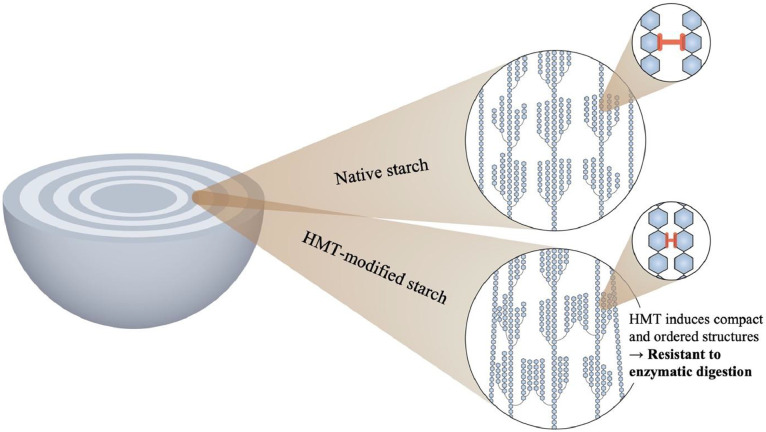
Schematic representation of the structural differences between native and HMT-modified starch.

HMT promotes the molecular realignment of amylose and amylopectin chains, leading to the formation of more compact and ordered double-helical structures that resist enzymatic hydrolysis (Li et al., 2020; Ma et al., 2020). This reorganization facilitates the development of RS within the starch matrix, as illustrated in Fig. 1. In the uncooked samples, including uncooked native starch, the high RS content is primarily associated with the intrinsic resistance of intact starch granules (Hayati et al., 2025). These granules possess a semi-crystalline lamellar architecture with densely packed crystalline domains, which restrict enzymatic accessibility. However, partial surface gelatinization or minor structural disruption during sample preparation may enhance enzymatic accessibility, thereby reducing RS levels. Notably, a substantial proportion of RS remains in the HMT-modified products after cooking, indicating the formation of heat-stable RS (Fonseca et al., 2021). This type of RS is attributed to the realignment of amylose and amylopectin chains into partially crystalline molecular structures that persist even after thermal processing (Ma et al., 2020).

Moisture is a key factor influencing RS formation during HMT. Sufficient moisture facilitates molecular mobility by allowing water to act as a plasticizer, promoting hydrogen bond formation between starch chains and supporting the development of crystalline structures that contribute to enzymatic resistance (Li et al., 2011). The present findings indicate that HMT at a moisture content of 20% (HMT-20) is the most effective in enhancing the RS content after cooking because this level provides the optimal balance between molecular mobility and structural stability. In contrast, moisture levels exceeding this threshold (i.e., in HMT-25 and HMT-30) result in a decreased RS content, potentially due to excessive gelatinization and the disruption of intra- and intermolecular interactions critical for RS formation. These findings are consistent with previous studies on lily, sweet potato, maize, wheat, and breadfruit starch, which have demonstrated that moderate moisture levels favor RS formation, while excessive moisture impairs granule integrity and reduces the content of RS (Huang et al., 2016; Li et al., 2020; Tan et al., 2017; Wang et al., 2016).

Although both the native and HMT-modified products in the uncooked samples exhibit a high RS content, these values are of limited biological relevance, as uncooked starch possesses a compact crystalline structure that restricts microbial fermentation (Xu et al., 2016). In contrast, RS that forms or remains after cooking, particularly in HMT-modified products, is more structurally accessible to gut microbiota due to partial disruption of granular crystallinity and reorganization of starch chains during thermal processing. As a result, RS that remains after cooking more closely reflects the physicochemical characteristics of starch encountered during feed processing and subsequent gastrointestinal digestion, making it more relevant for evaluating prebiotic potential in poultry feed. Given its comparatively high RS content and thermal stability, cooked HMT-20 was selected for subsequent in vitro fermentation studies to assess its prebiotic efficacy. The enhanced microbial accessibility and fermentability of cooked HMT-20 are discussed further in Section 3.2.

### 3.2 Evaluation of prebiotic potential

**Fig. 2:**
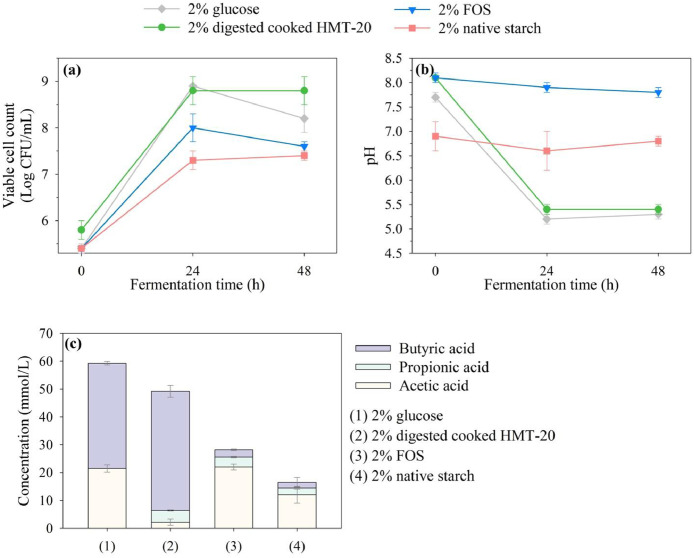
(a) Growth profiles, (b) pH changes, and (c) SCFA production by *Limosilactobacillus reuteri* TBRC291 in media supplemented with various carbon sources.

The growth profiles, pH changes, and SCFA production of *L. reuteri* in media supplemented with various carbon sources are presented in Fig. 2a–c, respectively, and the corresponding statistical analyses are provided in Supplementary Tables S1–3. The carbon sources included 2% glucose (control), FOS, and digested cooked HMT-20, representing a purified RS sample. Additionally, 2% native starch (uncooked sample) was used to compare the bacterial responses between the cooked and uncooked samples. The maximum bacterial counts were observed at 24 h, with values of 8.9, 8.0, 8.8, and 7.4 log CFU/mL for 2% glucose, FOS, digested cooked HMT-20, and native starch, respectively (Fig. 2a). These findings indicate that within the *L. reuteri* in vitro fermentation model, digested cooked HMT-20 supports bacterial proliferation to an extent comparable to that of FOS, a well-established reference prebiotic, suggesting its potential for use as a novel RS-based prebiotic. Moreover, bacterial growth was sustained up to 48 h with cooked HMT-20, which may be associated with the slower degradation rate of RS, providing a stable carbon source over a prolonged period for microbial viability (Fonseca et al., 2021).

A significant improvement in bacterial growth was observed with digested cooked HMT-20 compared to native starch, indicating enhanced bacterial accessibility and fermentability after thermal treatment. The substantial decrease in pH after 24 h of fermentation with digested cooked HMT-20 (Fig. 2b) further supports active bacterial metabolism and organic acid production. Correspondingly, according to the SCFA analysis (Fig. 2c), fermentation using digested cooked HMT-20 results in a significantly higher total SCFA concentration (49.2 mM) compared to native starch (16.4 mM) in the *L. reuteri* in vitro fermentation model. Among the SCFAs produced, butyric acid is predominant in the digested cooked HMT-20 sample. This observation is consistent with previous reports indicating that resistant starch fermentation is frequently associated with enhanced butyric acid production (Fuentes‐Zaragoza et al., 2011).

The increased butyric acid concentration observed in this study may be related to proposed RS fermentation pathways described in the literature, in which RS is hydrolyzed to glucose and subsequently metabolized via glycolysis to pyruvate, followed by conversion to acetyl-CoA and downstream SCFA biosynthesis. In this context, butyric acid formation has been attributed to the activity of butyryl-CoA:acetate CoA-transferase (BUT) and butyrate kinase (BUK) pathways (Vidrine et al., 2014; Wang et al., 2022). However, these mechanistic explanations remain hypothetical in the present study, as direct enzymatic or metabolic flux measurements were not performed.

In contrast, native starch exhibits the lowest SCFA levels, which may be attributed to its crystalline structure that limits enzymatic hydrolysis and microbial access. According to Le Blay et al. (2003), this structural resistance leads to a two-phase fermentation process that begins at the granule surface and gradually progresses toward the interior. This limited and delayed fermentation ultimately results in reduced SCFA production. Taken together, these findings indicate that, within the constraints of the *L. reuteri* in vitro fermentation model, cooked HMT-20 supports greater microbial fermentative activity, accompanied by lower pH and higher SCFA production, compared with native starch. These fermentation outcomes are consistent with the higher RS content of the cooked HMT-20 sample and support its selection for in vitro fermentation analysis based on the RS content results presented in Section 3.1.

### 3.3 Determination of optimal concentration of HMT-modified products in chicken feed

**Fig. 3:**
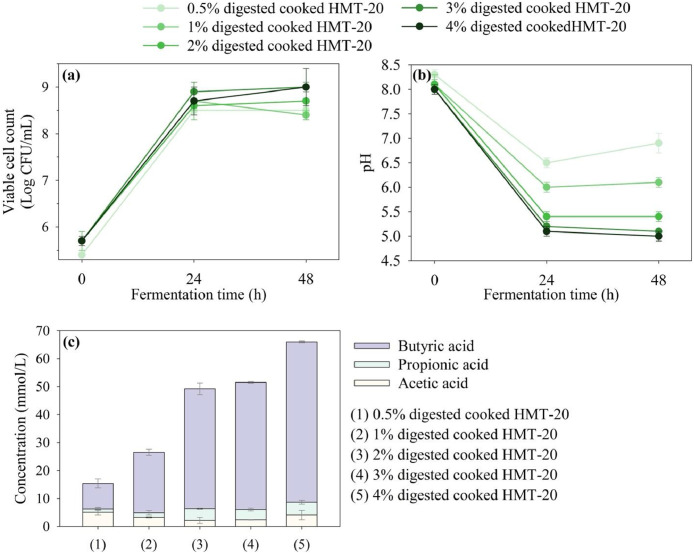
(a) Growth profiles, (b) pH changes, and (c) SCFA production by *Limosilactobacillus reuteri* TBRC291 in media supplemented with different doses of HMT-modified product.

To determine the optimal concentration of the HMT-modified products for inclusion in chicken feed, various concentrations (0.5, 1, 2, 3, and 4%) of the digested cooked HMT-20, representing pure RS, were evaluated. The growth profiles, pH changes, and SCFA production of *L. reuteri* in culture media enriched with digested cooked HMT-20 at these concentrations are shown in Figs. 3a–c, respectively. The corresponding statistical analyses are provided in Supplementary Tables S4–6.

The growth profiles for all concentrations show the highest bacterial growth at 24 h (range: log 8.5–8.9 CFU/mL), with no significant differences (*p* > 0.05) among the concentrations (Fig. 3a), and remain stable up to 48 h. In contrast, the pH changes after 24 h of fermentation vary depending on the concentration of digested cooked HMT-20 (Fig. 3b). At a concentration of 0.5%, the pH decreases to 6.5. Increasing the concentration to 1% results in a significant reduction to 6.0, while a further increase to 2% leads to a more pronounced decline to 5.4 (*p* < 0.05). However, increasing the concentration beyond 2% (to 3 and 4%) does not result in any further significant decrease in pH, with values ranging from 5.4 to 5.1. Thus, the results indicate that increasing the concentration of digested cooked HMT-20 beyond 2% does not significantly lower the pH. Notably, this trend contrasts with the SCFA production results (Fig. 3c). As the concentration of digested cooked HMT-20 increases from 0.5% to 1, 2, 3, and 4%, the SCFA levels increase significantly, rising from 15.4 to 26.4, 49.2, 51.6, and 66.0 mM, respectively. This can be explained by the buffering capacity of the medium and the type of SCFAs produced.

A 1% concentration of digested cooked HMT-20 was identified as the most appropriate for further investigation based on integrated biological and practical criteria. At this concentration, the pH of the medium reaches 6.0, which lies within the optimal pH range for *L. reuteri* growth (approximately 5.8–6.2) (Raskoshnaya et al., 2015) and closely reflects the physiological pH of the chicken cecum (approximately 5.5–7.0) (Deutschlander, 2024). This level also supports robust bacterial growth and leads to substantial SCFA production compared with other tested concentrations. Additionally, 1% digested cooked HMT-20 (equivalent to approximately 1% pure RS) aligns with the typical prebiotic inclusion levels in broiler diets (0.5–1.5%), as reviewed by Teng and Kim (2018), thereby supporting its economic and practical feasibility. Accordingly, considering its support of bacterial growth, maintenance of an optimal and physiologically relevant pH, stimulation of SCFA production, and practical applicability in broiler diets, this concentration was selected for subsequent experimental trials.

### 3.4 Diet formulation and RS content in experimental diets

The ingredients, chemical compositions, and RS contents of the experimental diets are presented in Table 1. The CON group received a control diet without the HMT-modified product, while the HMT-Feed group was given a diet supplemented with the cooked HMT-modified product (cooked HMT-20). Based on an in vitro starch digestibility analysis, the cooked HMT-20 (prepared via HMT followed by cooking) contained 20.7% RS. To achieve a dietary RS level of approximately 1%, the cooked HMT-20 was included at 5% of the total feed. For comparison, cooked native cassava starch, containing 12.6% RS, was added to the control diet at the same inclusion level (5%), yielding an estimated RS content of 0.62% in the CON group. Accordingly, the RS content in the CON and HMT-Feed diets was approximately 0.62% and 1.04%, respectively.

### 3.5 Growth performance

The growth performance parameters of the CON and HMT-Feed groups over the 35-day experimental period are summarized in Table 3. During days 1–14 (starter phase), broilers in the HMT-Feed group showed significantly lower BW and ADG (*p* < 0.05) at similar ADFI, resulting in a higher FCR (*p* < 0.05). This early growth depression may reflect reduced nutrient utilization, potentially associated with physiological immaturity and nutrient dilution. The inclusion of the HMT-modified product, characterized by a higher RS content, may have reduced digestible nutrient availability, thereby lowering dietary energy density (Slama et al., 2019). In young chicks with limited capacity to compensate through increased feed intake, this reduction in available energy could suppress weight gain and worsen FCR. Moreover, the immature digestive system and gut microbiota during the early post-hatch period may limit effective RS digestion and fermentation. The physicochemical properties of HMT-modified product may also partly increase intestinal viscosity, thereby reducing the diffusion of endogenous enzymes into the digesta and limiting their interaction with brush border membrane enzymes (Jha & Mishra, 2021), ultimately decreasing nutrient digestion and utilization.

**Table 3:** Growth performance parameters of CON and HMT-Feed groups.

Item		Group		SEM	*p*-value
		CON	HMT-Feed		
BW (g/bird)					
	1 day	38.6	38.6	0.035	0.816
	14 days	427	383	7.896	0.003
	21 days	851	770	15.25	0.003
	28 days	1352	1316	25.36	0.339
	35 days	1974	2031	29.29	0.180
ADG (g/bird)					
	1–14 days	27.8	24.6	0.564	0.003
	15–21 days	60.5	55.3	1.483	0.027
	22–28 days	71.5	78.0	2.270	0.083
	29–35 days	88.9	102	2.489	0.003
	1–35 days	55.3	56.9	1.011	0.276
ADFI (g/bird)					
	1–14 days	34.6	36.5	1.615	0.421
	15–21 days	76.5	70.7	1.718	0.038
	22–28 days	117	117	1.602	0.981
	29–35 days	156	159	4.122	0.639
	1–35 days	83.8	84.0	1.370	0.931
FCR					
	1–14 days	1.25	1.49	0.068	0.033
	15–21 days	1.26	1.28	0.012	0.389
	22–28 days	1.64	1.52	0.043	0.100
	29–35 days	1.76	1.56	0.049	0.015
	1–35 days	1.52	1.48	0.020	0.183

HMT= heat-moisture treatment; CON= control group, received feed without the HMT-modified product; HMT-Feed= treatment group, given feed containing the HMT-modified product; BW= body weight; ADG= average daily weight gain; ADFI= average daily feed intake; FCR= feed conversion ratio; SEM= standard error of the mean. Data were analyzed using Student’s *t*-test. When Levene’s test indicated unequal variances (*p* < 0.05), Welch’s *t*-test was used.

During days 15–21, broilers in the HMT-Feed group showed significantly lower BW, ADG, and ADFI (*p* < 0.05) without differences in FCR, indicating that the growth suppression during this period was primarily intake-driven rather than due to impaired feed efficiency. No significant differences in BW, ADG, ADFI, or FCR were observed during days 22–28 (*p* > 0.05). However, during days 29–35 (finisher phase), the HMT-Feed group exhibited significantly greater ADG and lower FCR (*p* < 0.05), with no differences in ADFI or BW (*p* > 0.05). The improved performance during the finisher phase may reflect physiological adaptation, including enhanced digestive enzyme production (Yadav et al., 2019), increased digestive tract development (González-Alvarado et al., 2008), and alterations in microbial composition and activity (CK Rajendran et al., 2017). As birds mature, gastrointestinal development and a more established hindgut microbiota likely enhance RS fermentation and SCFA production, thereby providing additional energy for enterocytes. This interpretation is supported by the higher villus-to-crypt ratio and total SCFA concentration observed on day 35 in the HMT-Feed group (as presented in 3.8, 3.9).

Notably, no differences in overall BW, FI, ADG, or FCR were detected across days 1–35. This outcome may be explained by compensatory growth, whereby early growth suppression is followed by accelerated growth, enabling birds to achieve body weights comparable to those whose growth was not previously restricted (Radulovic et al., 2021). However, these interpretations are based on indirect physiological indicators, and further studies incorporating direct measurements of nutrient digestibility, digestive enzyme activity, and gut morphology dynamics are warranted to confirm the proposed mechanisms. Collectively, these findings suggest that the HMT-modified product may serve as a functional fiber source in broiler diets without compromising overall growth performance under the experimental conditions.

### 3.6 Carcass traits

The carcass traits are summarized in Table 4. The most notable effect of prebiotic supplementation with the HMT-modified product is a significant reduction in abdominal fat (*p* < 0.001). Specifically, abdominal fat content decreased from 1.03% in the CON group to 0.78% in the HMT-Feed group. This finding is consistent with previous reports showing reduced fat deposition following prebiotic or probiotic supplementation (Abdel-Hafeez et al., 2016) and aligns with earlier evidence that RS can attenuate abdominal fat accumulation in animal models (Vidrine et al., 2014). Broilers receiving RS-containing diets exhibited not only a lower abdominal fat percentage but also smaller adipocyte diameters in abdominal adipose tissue, indicating reduced adipocyte hypertrophy rather than simple redistribution of lipid stores (Zhang et al., 2020b). These phenotypic responses were accompanied by reduced expression of lipogenic regulatory genes, such as fatty acid synthase (FAS) and acetyl-CoA carboxylase (ACC), along with increased expression of genes involved in fatty acid oxidation, including peroxisome proliferator-activated receptor alpha (PPARα) and phosphoenolpyruvate carboxykinase (PEPCK) (Liu et al., 2020b). These findings are also consistent with the growth performance pattern observed in the present study, where early growth suppression was followed by compensatory growth without affecting final body weight. Similar responses have been reported in growth-modulating or nutrient dilution strategies that reduce fat deposition while maintaining market weight (Radulovic et al., 2021), suggesting that the reduced abdominal fat in the HMT-Feed group may be metabolically associated with altered nutrient partitioning during early growth rather than an overall limitation of growth potential.

**Table 4:** Carcass traits and meat quality characteristics of CON and HMT-Feed groups.

Parameter			Group		SEM	*p*-value
			CON	HMT-Feed		
Carcass traits						
		Slaughter weight (g)	1974	2031	29.29	0.180
		HCW (g)	1523	1570	24.46	0.169
		Feet (%HCW)	5.05	5.01	0.084	0.727
		Head (%HCW)	2.68	2.64	0.042	0.518
		Spleen (%HCW)	0.11	0.10	0.003	0.292
		Abdominal fat (%HCW)	1.03	0.78	0.042	<0.001
		GI (%HCW)	10.3	10.3	0.129	0.974
		CCW (g)	1475	1533	24.23	0.094
		Dressing out (%)	74.6	75.4	0.308	0.092
		Heart (%CCW)	0.78	0.74	0.013	0.028
		Liver (%CCW)	2.55	2.54	0.041	0.869
		Wings (%CCW)	9.56	9.39	0.096	0.241
		Breast (%CCW)	24.2	25.7	0.251	<0.001
		Thighs (%CCW)	30.0	28.6	0.257	<0.001
		Meat/bone ratio	7.21	6.99	0.161	0.352
Meat quality characteristics						
	Meat color					
		L*	57.1	56.1	0.485	0.153
		a*	1.56	1.32	0.129	0.204
		b*	4.33	3.46	0.228	0.008
	pH					
		pH_45_	5.97	6.07	0.028	0.014
		pH_u_	5.80	5.82	0.013	0.137
	WHC (%)					
		Drip loss	3.70	3.56	0.143	0.495
		Thawing loss	6.06	5.36	0.313	0.114
		Cooking loss	33.6	32.2	0.306	0.002

HMT= heat-moisture treatment; CON= control group, received feed without the HMT-modified product; HMT-Feed= treatment group, given feed containing the HMT-modified product; HCW= hot carcass weight; GI= gastrointestinal tract; CCW= cold carcass weight; L*= lightness; a*= redness; b*= yellowness; pH_45_= pH of meat at 45 min after slaughtering; pH_u_= pH of meat at 24 h after slaughtering; WHC= water-holding capacity; SEM= standard error of the mean. Data were analyzed using Student’s *t*-test. When Levene’s test indicated unequal variances (*p* < 0.05), Welch’s *t*-test was used.

In addition to the reduction in abdominal fat, the relative heart weight (%CCW) is significantly reduced (*p* < 0.05), declining from 0.78% in the CON group to 0.74% in the HMT-Feed group. Increased abdominal fat has been associated with greater cardiac weight, which may result from cardiac hypertrophy, hypertension, and insulin resistance (Neeland et al., 2013). Interestingly, the HMT-modified product does not significantly affect the hot and cold carcass yield, gastrointestinal tract weight, or other evaluated carcass parameters (*p* > 0.05), suggesting that its primary prebiotic effect is related to fat metabolism rather than overall organ development or carcass composition. In terms of the carcass portions, the HMT group exhibits a significantly higher breast yield (25.7%) compared to the CON group (24.2%; *p* < 0.05), which is attributed to improved villus growth and protein absorption in the HMT group. Additionally, Tavaniello et al. (2018) reported that prebiotic-fed broilers demonstrated a larger muscle fiber diameter, which has been associated with an increased breast yield. However, the thigh yield significantly decreases, from 30.0% to 28.6% (*p* < 0.05). This shift might result from the reduced lipogenesis observed in the HMT group, inducing a lower fat content in the thigh muscle (Ibrahim et al., 2021).

### 3.7 Meat quality characterization

The meat quality parameters of the breast samples from the CON and HMT-Feed groups are presented in Table 4. No significant difference was observed in L* (lightness), with mean values of 57.1 and 56.1 for the CON and HMT-Feed groups, respectively. These results indicate that the HMT-Feed does not adversely affect the visual appearance of breast meat in terms of lightness. However, the HMT-Feed group shows a significantly lower b* (yellowness), possibly caused by a reduction in lipogenesis and lipid accumulation in the breast muscle of broilers fed with a prebiotic-supplemented diet (Ibrahim et al., 2021).

In terms of pH, a significantly lower pH_45_ value was observed in the CON group (5.97) compared to the HMT-Feed group (6.07; *p* < 0.05). Following slaughter, the pH of meat typically declines as lactic acid accumulates through anaerobic glycolysis (Pearce et al., 2011). A slightly higher pH_45_ in the HMT-Feed group suggests a slower rate of postmortem glycolysis or a potential effect of the prebiotic treatment on muscle energy metabolism. Although the difference is modest, it may have implications for early postmortem muscle biochemistry and initial meat quality traits. However, the pH_u_ of the HMT-Feed group (5.80) is not significantly different from that of the CON group (5.82). All values remained within the pH range (approximately 5.7–6.2) considered indicative of good-quality broiler meat for commercial production (Gumus & Gelen, 2023).

The WHC of meat is a critical quality attribute that directly influences product yield, processing performance, and consumer acceptability (Cheng & Sun, 2008). In the present study, while no significant differences were observed in the drip or thawing loss between the groups, the cooking loss is significantly lower in the HMT-Feed group (32.2%) compared to the CON group (33.6%; *p* < 0.05). This reduction may be associated with the improved functional properties of muscle tissue influenced by prebiotic supplementation. These findings are consistent with the results of Mohammed and Kareem (2022), who reported that the dietary inclusion of prebiotics can reduce cooking loss in poultry meat.

### 3.8 Gastrointestinal tract characterization and histological structure analysis

The effects of the HMT-modified product on the relative length and morphology of the small intestine in broilers at day 35 are presented in Table 5, and representative images of the small intestine are shown in Supplementary Fig. S1. No significant differences were observed in the relative lengths of the duodenum, jejunum, and ileum between the HMT-Feed and CON groups (*p* > 0.05). This contrasts with the results of Zhang et al. (2020a), who reported that dietary supplementation with corn-derived RS significantly increases the relative lengths of all small intestinal segments (*p* < 0.05). The discrepancy may be attributed to differences in the type and physicochemical properties of RS sources (Jha & Mishra, 2021), the level of inclusion, or the duration of dietary exposure. Despite the lack of effects on intestinal length, a significant increase in the villus height was observed in the duodenum, jejunum, and ileum of the HMT-Feed group (*p* < 0.05), suggesting enhanced mucosal development and absorptive capacity in the distal intestine (Wijtten et al., 2012). However, studies have reported that high levels of RS in broiler diets can lead to a linear reduction in the villus height by 42 days of age (Liu et al., 2020a). Interestingly, all segments of the small intestine exhibit significantly lower crypt depths in the HMT-Feed group compared to the CON group (*p* < 0.05), indicating reduced epithelial turnover and possibly improved gut integrity (Montagne et al., 2003). This may be associated with RS reaching the large intestine and undergoing bacterial fermentation to SCFAs, particularly butyrate, a major energy source for colonocytes with limited systemic availability (Vidrine et al., 2014), consistent with the increased butyric acid concentration observed in the present study. Consequently, the villus-to-crypt ratio is significantly higher in all small intestinal segments of the HMT-Feed group (*p* < 0.05). This ratio is widely recognized as a reliable indicator of the functional capacity of the small intestine, particularly in relation to digestion and nutrient absorption. Further, it is influenced by several factors, including microbial compositions, SCFA concentrations, and intestinal pH, all of which can modulate epithelial cell proliferation, maintain mucosal integrity, and affect the overall digestive efficiency (Pan & Yu, 2014; Singh & Kim, 2021). A higher ratio generally reflects an enhanced absorptive capacity and improved intestinal functioning. Therefore, the findings of the present study further confirm the beneficial effects of the HMT-modified product on intestinal morphology and function. These structural improvements may enhance nutrient absorption, ultimately contributing to the improved overall health of broilers.

**Table 5:** Gastrointestinal tract characteristics and histological structure of CON and HMT-Feed groups.

Parameter			Group		SEM	*p*-value
			CON	HMT-Feed		
Gastrointestinal tract length (cm/kg BW)						
	Duodenum		14.2	13.7	0.27	0.200
	Jejunum		34.9	34.9	0.85	0.997
	Ileum		36.2	36.8	0.96	0.640
Gastrointestinal tract histology						
	Duodenum					
		Villus height (µm)	1004	1321	67.7	0.006
		Crypt depth (µm)	262	174	11.7	<0.001
		Villus-to-crypt ratio	4.04	7.91	0.30	<0.001
	Jejunum					
		Villus height (µm)	808	950	39.9	0.021
		Crypt depth (µm)	172	104	8.45	<0.001
		Villus-to-crypt ratio	5.01	9.59	0.29	<0.001
	Ileum					
		Villus height (µm)	405	530	16.9	<0.001
		Crypt depth (µm)	113	71.8	4.43	<0.001
		Villus-to-crypt ratio	3.61	7.86	0.23	<0.001

HMT= heat-moisture treatment; CON= control group, received feed without the HMT-modified product; HMT-Feed= treatment group, given feed containing the HMT-modified product; SEM= standard error of the mean. Data were analyzed using Student’s *t*-test. When Levene’s test indicated unequal variances (*p* < 0.05), Welch’s *t*-test was used.

### 3.9 Cecal pH and cecal fermentation-derived SCFAs

The results relating to the cecal pH and fermentation-derived SCFAs at day 35 in the CON and HMT-Feed groups are presented in Table 6. The cecal pH in the HMT-Feed group is significantly lower than that of the CON group (*p* < 0.05). However, the pH values in both groups remain within the normal physiological range for the chicken cecum: approximately 5.5–7.0 (Asare et al., 2021). The reduction in the cecal pH suggests increased microbial fermentation activity, potentially due to the prebiotic effects of the HMT-modified product.

**Table 6:** Cecal pH and cecal fermentation-derived SCFAs on day 35 of CON and HMT-Feed groups.

Parameter		Group		SEM	*p*-value
		CON	HMT-Feed		
Cecal pH		6.34	5.95	0.120	0.037
Cecal fermentation-derived SCFAs (μmol/g)					
	Acetic acid	227	281	21.38	0.086
	Propionic acid	18.9	22.7	2.263	0.274
	Butyric acid	54.9	81.6	5.411	0.001
	Total SCFAs	301	384	25.08	0.026

HMT= heat-moisture treatment; CON= control group, received feed without the HMT-modified product; HMT-Feed= treatment group, given feed containing the HMT-modified product; SCFA= short-chain fatty acid; SEM= standard error of the mean. Data were analyzed using Student’s *t*-test. When Levene’s test indicated unequal variances (*p* < 0.05), Welch’s *t*-test was used.

The production of SCFAs was further measured to assess the microbial fermentation process. The total SCFA content is significantly higher in the HMT-Feed group compared to the CON group (*p* < 0.05), with a notable increase in butyric acid. In contrast, the concentrations of acetic acid and propionic acid remain statistically unchanged between the two groups (*p* > 0.05). These findings are consistent with those of Oluseyifunmi et al. (2024), who reported an increased total SCFA production in broilers fed with RS-supplemented diets. Similarly, Akbaryan et al. (2019) found that the dietary inclusion of 1% RS does not significantly alter the concentrations of acetic acid and propionic acid. In both groups, acetic acid is the most abundant SCFA, followed by butyric and propionic acid. This pattern is consistent with previous studies indicating that acetic acid is typically the dominant SCFA in ceca (Deutschlander, 2024). Supporting these in vivo observations, the in vitro results of the present study further demonstrate that RS fermentation from the HMT-modified product leads to a significant reduction in pH and a marked increase in butyric acid production. These findings highlight the potential of RS to enhance the growth of beneficial bacteria and stimulate SCFA production; this may contribute to the suppression of pathogenic bacteria, maintenance of intestinal barrier integrity, and modulation of immune responses (Liu et al., 2021).

### 3.10 Cecal microbiota analysis

**Fig. 4:**
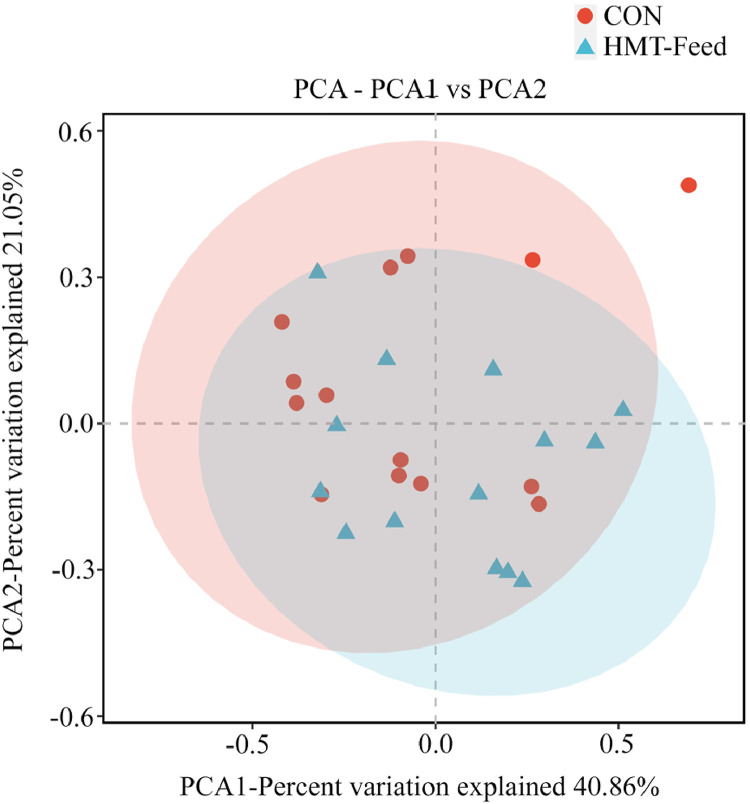
Beta diversity between the CON and HMT-Feed groups.

The beta diversity between the CON and HMT-Feed groups is shown in Fig. 4. No significant differences were observed between the groups (*p* > 0.05), indicating that the dietary inclusion of the HMT-modified product does not cause a substantial shift in the overall composition of the gut microbial community. Consistently, alpha diversity indices, including the Chao1, Shannon–Weaver, and Simpson indices, are presented in Table 7, with none of these metrics showing significant differences between the groups (*p* > 0.05). These findings suggest that the overall microbial richness or diversity is not markedly affected by the HMT-modified product.

**Table 7:** Alpha diversity indices (Chao1, Shannon–Weaver, and Simpson) for CON and HMT-Feed groups.

Parameter	Group		SEM	*p*-value
	CON	HMT-Feed		
Chao1 index	331	328	10.04	0.810
Shannon–Weaver index	5.42	5.55	0.092	0.323
Simpson index	0.93	0.94	0.009	0.461

HMT= heat-moisture treatment; CON= control group, received feed without the HMT-modified product; HMT-Feed= treatment group, given feed containing the HMT-modified product; SCFA= short-chain fatty acid; SEM= standard error of the mean. Data were analyzed using Student’s *t*-test. When Levene’s test indicated unequal variances (*p* < 0.05), Welch’s *t*-test was used.

**Fig. 5:**
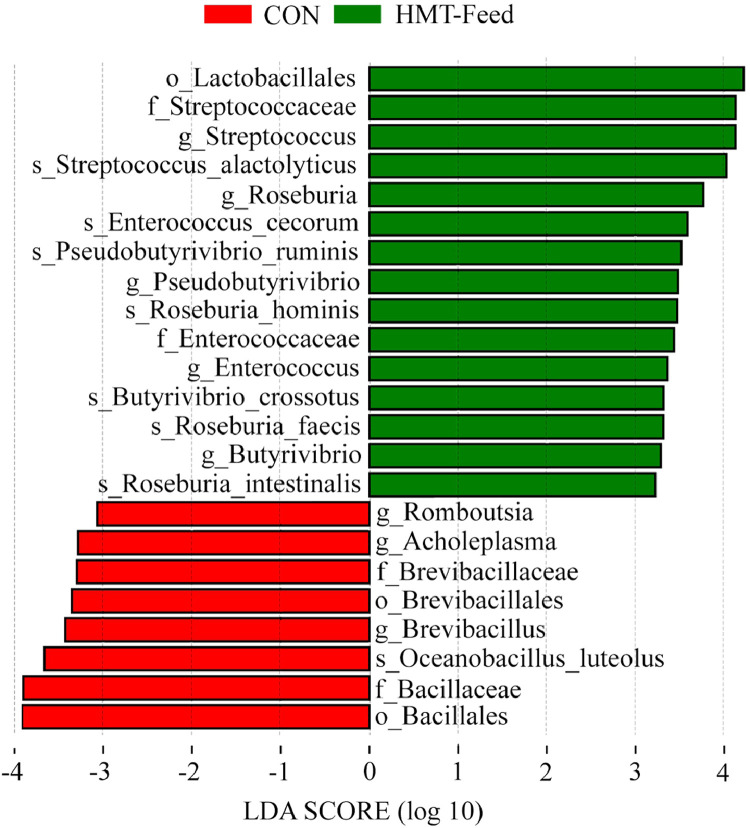
Differentially abundant bacterial taxa between the CON and HMT-Feed groups, identified via linear discriminant analysis effect size (LEfSe) analysis.

To provide detailed quantitative information on microbial composition, the relative abundances at the phylum and species levels, including specific taxa with significant differences between the CON and HMT-Feed groups, are presented in Supplementary Table S7. Despite the similarity in the overall diversity, LEfSe revealed significant differences in specific bacterial taxa between the two groups. As shown in Fig. 5, members of the order Lactobacillales, particularly *Streptococcus alactolyticus*, are significantly enriched in the HMT-Feed group (LDA score > 2.0, *p* < 0.05). This suggests that RS fermentation in the HMT-modified product strongly promotes the proliferation of lactic acid bacteria in the gut microbiota of chickens, especially those within Lactobacillales, which are widely recognized for their probiotic properties (Zhang et al., 2021). In addition to lactic acid bacteria, several well-established butyrate-producing bacteria are significantly more abundant in the HMT-Feed group, including the genera *Roseburia, Butyrivibrio*, and *Pseudobutyrivibrio*, along with their respective species (*Roseburia hominis, R. faecis, R. intestinalis, Butyrivibrio crossotus*, and *Pseudobutyrivibrio ruminis*; LDA score > 2.0, *p* < 0.05) (Pandit et al., 2018; Polansky et al., 2016; Rychlik, 2020). These taxa are known for their ability to degrade complex carbohydrates and ferment them into butyric acid. This observation is consistent with the cecal fermentation analysis, which indicated a significant increase in cecal butyrate levels, further confirming the prebiotic effect of the HMT-modified product in chickens.

Notably, the simultaneous enrichment of both lactic acid-producing bacteria (e.g., Lactobacillales) and butyrate producers suggests a synergistic modulation of the gut microbiota in chickens, supporting improved intestinal health and the well-being of the host.

## 4 Conclusion

The optimized HMT-modified product (HMT-20) exhibited an RS content of 20.7% in the cooked sample and demonstrated in vitro prebiotic potential by promoting probiotic growth, lowering pH, and increasing SCFA production, particularly butyric acid. In vitro screening identified 1% cooked HMT-20 as the optimal level for application in chicken feed.

In the in vivo study, these findings support the potential development of the HMT-modified product as a value-added and sustainable feed additive for the poultry industry. Supplementation with 1% cooked HMT-20 (HMT-Feed) had no adverse effects on the overall growth performance during the 35-day trial. Notably, during the finisher phase over days 29–35, broilers fed with the HMT-modified product showed a significantly greater ADG and an improved FCR, indicating potential benefits for chicken breeds or production systems with extended rearing periods (e.g., 42 days or more).

Regarding the carcass traits and meat quality, HMT-Feed reduced abdominal fat and increased breast weight, while thigh and heart weights were lower, and cooking loss was reduced. Improvements in intestinal morphology, particularly an increased villus height and a higher villus-to-crypt ratio, suggest an enhanced absorptive capacity and gut functionality. Furthermore, the HMT-Feed significantly reduced the cecal pH and increased total SCFA concentrations, especially butyric acid. Deep microbiota analysis revealed a favorable modulation of the gut microbial community, with the enrichment of lactic acid–producing bacteria (order Lactobacillales) and butyrate-producing bacteria, supporting improved gut health.

All experiments were conducted using cassava starch from a single production batch to ensure material consistency; however, this represents a limitation in terms of capturing potential compositional variability among different industrial batches. In addition, the in vitro fermentation model employed *L. reuteri* as a representative probiotic strain to verify functional potential, and further studies including mixed microbial communities would broaden understanding of prebiotic selectivity. Although an optimal inclusion level was identified through in vitro screening and applied in the in vivo study, evaluation of multiple dietary inclusion levels under commercial production conditions would strengthen practical applicability, particularly when combined with economic feasibility analyses to support large-scale application of HMT-modified products in the poultry industry.

## Data availability

The raw sequencing data in FASTQ format, comprising full-length 16S rRNA gene sequences of the chicken cecal microbiota, have been deposited in the Mendeley Data repository and are publicly available via the Digital Object Identifier (DOI): 10.17632/r839mtn9mj.2 (https://data.mendeley.com/datasets/r839mtn9mj/2).

## Declaration of generative AI and AI-assisted technologies in the manuscript preparation process

During the preparation of this work the authors used ChatGPT (OpenAI) in order to assist with grammar correction and English language editing in some parts of the manuscript. After using this tool, the authors reviewed and edited the content as needed and take full responsibility for the content of the published article.

## CRediT authorship contribution statement

**Watcharamon Prasert:** Writing – original draft, Validation, Methodology, Investigation, Formal analysis. **Nattaphong Akrimajirachoote:** Writing – review & editing, Writing – original draft, Validation, Supervision, Methodology, Investigation, Formal analysis. **Montri Pattarapanawan:** Writing – review & editing, Writing – original draft, Validation, Methodology, Investigation, Formal analysis, Conceptualization. **Attawit Kovitvadhi:** Writing – review & editing, Validation, Methodology, Investigation, Formal analysis. **Sukanya Phuengjayaem:** Writing – review & editing, Methodology, Formal analysis. **Haibo Qin:** Writing – review & editing, Methodology, Formal analysis. **Bing-Zheng Li:** Writing – review & editing, Methodology, Formal analysis. **Nitnipa Soontorngun:** Writing – review & editing. **Niyada Lansubsakul:** Investigation, Formal analysis. **Sathita Areerat:** Investigation. **Ditpon Kotatha:** Writing – review & editing, Writing – original draft, Visualization, Validation, Supervision, Project administration, Methodology, Investigation, Funding acquisition, Formal analysis, Conceptualization.

## Declaration of competing interest

Attawit Kovitvadhi is employed by KU Vet Innova Nutricare Co., Ltd., a company based at the Faculty of Veterinary Medicine, Kasetsart University. This affiliation does not constitute a conflict of interest related to the content of this study. The remaining authors declare that they have no conflict of interest.

## Appendix Supplementary materials

Image, application 1
