# Supplementary material for: Enhancing the resistant starch content of cassava starch via heat-moisture treatment for application as a prebiotic in chicken feed
Source: Vet Anim Sci. 2026 Mar 23;32:100630. doi: 10.1016/j.vas.2026.100630 (PMC13068798; doi:10.1016/j.vas.2026.100630)
Supplement: Supplementary file 1 [file mmc1.docx]

**Enhancing the resistant starch content of cassava starch via heat-moisture treatment for application as a prebiotic in chicken feed**

**Supplementary Table S1** Viable cell counts of *Limosilactobacillus reuteri* TBRC291 cultivated in MRS and modified MRS media, with the carbon source replaced by 2% glucose, 2% digested cooked HMT-20, 2% FOS, and 2% native starch.

| Viable cell count (log CFU/mL) | | | |
| --- | --- | --- | --- |
| Sample | Fermentation time (h) | | |
|  | 0 h | 24 h | 48 h |
| 2% glucose | 5.4 ± 0.1^bC^ | 8.9 ± 0.1^aA^ | 8.1 ± 0.2^bB^ |
| 2% digested cooked HMT-20 | 5.6 ± 0.2^aB^ | 8.6 ± 0.3^aA^ | 8.7 ± 0.4^aA^ |
| 2% FOS | 5.4 ± 0.0^bB^ | 7.9 ± 0.2^bA^ | 7.6 ± 0.1^cA^ |
| 2% native starch | 5.4 ± 0.0^bB^ | 7.4 ± 0.1^cA^ | 7.3 ± 0.0^cA^ |

HMT = heat-moisture treatment; FOS = fructo-oligosaccharides. Lowercase letters (a, b, …) indicate a significant difference (*p* < 0.05) among samples with the same incubation period. Uppercase letters (A, B, …) indicate a significant difference (*p* < 0.05) among incubation periods in the same medium.

**Supplementary Table S2** pH changes of *L. reuteri* cultivated in MRS and modified MRS media, with the carbon source replaced by 2% glucose, 2% digested cooked HMT-20, 2% FOS, and 2% native starch.

| pH | | | |
| --- | --- | --- | --- |
| Sample | Fermentation time (h) | | |
|  | 0 h | 24 h | 48 h |
| 2% glucose | 7.7 ± 0.1^bA^ | 5.2 ± 0.1^cB^ | 5.3 ± 0.1^cB^ |
| 2% digested cooked HMT-20 | 8.1 ± 0.1^aA^ | 5.4 ± 0.1^cB^ | 5.4 ± 0.1^cB^ |
| 2% FOS | 8.1 ± 0.1^aA^ | 7.9 ± 0.1^aB^ | 7.8 ± 0.1^aB^ |
| 2% native starch | 6.9 ± 0.3^cA^ | 6.6 ± 0.4^bA^ | 6.8 ± 0.1^bA^ |

HMT = Heat-moisture treatment; FOS = fructo-oligosaccharides. Lowercase letters (a, b, …) indicate a significant difference (*p* < 0.05) among samples with the same incubation period. Uppercase letters (A, B, …) indicate a significant difference (*p* < 0.05) among incubation periods in the same medium.

**Supplementary Table S3** SCFA production of *L. reuteri* cultivated in MRS and modified MRS media, with the carbon source replaced by 2% glucose, 2% digested cooked HMT-20, 2% FOS, and 2% native starch.

| Sample | Acetic acid | Propionic acid | Butyric acid | Total SCFAs |
| --- | --- | --- | --- | --- |
|  | mmol/L | | | |
| 2% glucose | 21.5 ± 1.3^a^ | 0^c^ | 37.8 ± 0.6^b^ | 59.3 ± 0.7^a^ |
| 2% digested cooked HMT-20 | 2.2 ± 1.1^c^ | 4.2 ± 0.2^a^ | 42.8 ± 2.1^a^ | 49.2 ± 1.9^b^ |
| 2% FOS | 22.0 ± 1.1^a^ | 3.6 ± 0.3^a^ | 2.6 ± 0.3^c^ | 28.2 ± 0.9^c^ |
| 2% native starch | 12.0 ± 3.0^b^ | 2.5 ± 0.4^b^ | 2.0 ± 1.7^c^ | 16.4 ± 4.4^d^ |

HMT = Heat-moisture treatment; FOS = fructo-oligosaccharides; SCFA = short-chain fatty acid. Lowercase letters (a, b, …) indicate significant differences (*p* < 0.05) among samples within the same column.

**Supplementary** **Table S4** Viable cell counts of *L. reuteri* cultivated in modified MRS media with the carbon source replaced by HMT-modified products with different concentrations.

| Viable cell counts (log CFU/mL) | | | |
| --- | --- | --- | --- |
| Sample | Fermentation time (h) | | |
|  | 0 h | 24 h | 48 h |
| 0.5% digested cooked HMT-20 | 5.4 ± 0.0^bB^ | 8.5 ± 0.1^aA^ | 8.5 ± 0.1^abA^ |
| 1% digested cooked HMT-20 | 5.7 ± 0.1^aB^ | 8.7 ± 0.2^aA^ | 8.4 ± 0.1^cA^ |
| 2% digested cooked HMT-20 | 5.6 ± 0.2^aB^ | 8.6 ± 0.3^aA^ | 8.7 ± 0.4^abA^ |
| 3% digested cooked HMT-20 | 5.7 ± 0.1^aB^ | 8.9 ± 0.2^aA^ | 9.0 ± 0.1^aA^ |
| 4% digested cooked HMT-20 | 5.7 ± 0.1^aB^ | 8.7 ± 0.3^aA^ | 9.0 ± 0.4^aA^ |

HMT = Heat-moisture treatment. Lowercase letters (a, b, …) indicate a significant difference (*p* < 0.05) among samples with the same incubation period. Uppercase letters (A, B, …) indicate a significant difference (*p* < 0.05) among incubation periods in the same medium.

**Supplementary** **Table S5** pH changes of *L. reuteri* cultivated in modified MRS media with the carbon source replaced by HMT-modified products with different concentrations.

| pH | | | |
| --- | --- | --- | --- |
| Sample | Fermentation time (h) | | |
|  | 0 h | 24 h | 48 h |
| 0.5% digested cooked HMT-20 | 8.3 ± 0.1^aA^ | 6.5 ± 0.1^aC^ | 6.9 ± 0.2^aB^ |
| 1% digested cooked HMT-20 | 8.1 ± 0.2^abA^ | 6.0 ± 0.1^bB^ | 6.1 ± 0.1^bB^ |
| 2% digested cooked HMT-20 | 8.1 ± 0.1^abA^ | 5.4 ± 0.1^cB^ | 5.4 ± 0.1^cB^ |
| 3% digested cooked HMT-20 | 8.0 ± 0.1^bA^ | 5.2 ± 0.1^cdB^ | 5.1 ± 0.0^dB^ |
| 4% digested cooked HMT-20 | 8.0 ± 0.1^bA^ | 5.1 ± 0.1^dB^ | 5.0 ± 0.0^dB^ |

HMT = heat-moisture treatment. Lowercase letters (a, b, …) indicate a significant difference (*p* < 0.05) among samples with the same incubation period. Uppercase letters (A, B, …) indicate a significant difference (*p* < 0.05) among incubation periods in the same medium.

| Sample | Acetic acid | Propionic acid | | Butyric acid | Total SCFAs |
| --- | --- | --- | --- | --- | --- |
|  | mmol/L | | | | |
| 0.5% digested cooked HMT-20 | 5.1 ± 1.0^a^ | 1.2 ± 0.5^b^ | 9.1 ± 1.6^e^ | | 15.4 ± 1.6^d^ |
| 1% digested cooked HMT-20 | 3.3 ± 0.2^ab^ | 1.6 ± 0.8^b^ | 21.6 ± 1.1^d^ | | 26.4 ± 1.4^c^ |
| 2% digested cooked HMT-20 | 2.2 ± 1.1^b^ | 4.2 ± 0.2^a^ | 42.8 ± 2.1^c^ | | 49.2 ± 1.9^b^ |
| 3% digested cooked HMT-20 | 2.4 ± 0.1^b^ | 3.7 ± 0.5^a^ | 45.4 ± 0.3^b^ | | 51.6 ± 0.1^b^ |
| 4% digested cooked HMT-20 | 4.1 ± 1.7^ab^ | 4.6 ± 0.7^a^ | 57.3 ± 0.3^a^ | | 66.0 ± 2.7^a^ |

**Supplementary** **Table S6** SCFAs production of *L. reuteri* cultivated in modified MRS media with the carbon source replaced by HMT-modified products with different concentrations.

HMT = Heat-moisture treatment; SCFA = short-chain fatty acid. Lowercase letters (a, b, …) indicate significant differences (*p* < 0.05) among samples within the same column.

**Supplementary Table S7** Relative abundance on phylum, species level, specific bacteria and significant results between CON and HMT-Feed group

| Taxonomy | | Groups | | SEM | *p*-value |
| --- | --- | --- | --- | --- | --- |
|  |  | CON | HMT-Feed |  |  |
| Phylum level | |  |  |  |  |
|  | Firmicutes | 65.0 | 67.3 | 2.054 | 0.432 |
|  | Bacteroidetes | 32.7 | 31.4 | 1.826 | 0.619 |
|  | Proteobacteria | 0.85 | 1.08 | 0.204 | 0.429 |
|  | Verrucomicrobiota | 1.39 | 0.11 | 0.546 | 0.228 |
|  | Unclassified | 0.03 | 0.03 | 0.003 | 0.773 |
|  | Desulfobacterota | 0.00 | 0.05 | 0.013 | 0.064 |
|  | Fusobacteriota | 0.00 | 0.00 | 0.001 | 0.747 |
|  | Deferribacterota | 0.00 | 0.00 | 0.001 | 0.658 |
|  | Campilobacterota | 0.00 | 0.00 | 0.000 | 1.000 |
|  | Actinobacteriota | 0.01 | 0.01 | 0.002 | 0.689 |
|  | Firmicutes/Bacteroidetes ratio | 2.08 | 2.41 | 0.245 | 0.377 |
| Total *Lactobacillus* sp. | | 1.32 | 1.79 | 0.382 | 0.417 |
| Species level (Top 20) | |  |  |  |  |
|  | *Bacteroides fragilis* | 17.4 | 14.2 | 1.994 | 0.277 |
|  | *Parabacteroides merdae* | 12.2 | 13.7 | 2.567 | 0.670 |
|  | *Coprococcus comes* | 3.34 | 2.83 | 0.284 | 0.220 |
|  | *Flavonifractor plautii* | 3.06 | 2.78 | 0.418 | 0.644 |
|  | *Blautia coccoides* | 2.01 | 2.81 | 0.363 | 0.133 |
|  | *Butyricicoccus pullicaecorum* | 2.06 | 2.18 | 0.215 | 0.721 |
|  | *Blautia producta* | 1.76 | 2.15 | 0.203 | 0.189 |
|  | *Eisenbergiella tayi* | 1.81 | 2.01 | 0.254 | 0.589 |
|  | *Intestinimonas butyriciproducens* | 1.81 | 1.92 | 0.155 | 0.637 |
|  | *Streptococcus alactolyticus* | 0.73 | 2.73 | 0.432 | 0.010 |
|  | *Anaerotruncus colihominis* | 1.76 | 1.68 | 0.123 | 0.661 |
|  | *Hungatella hathewayi* | 1.58 | 1.60 | 0.139 | 0.920 |
|  | *Erysipelatoclostridium ramosum* | 1.47 | 1.34 | 0.287 | 0.758 |
|  | *Ruminococcus bromii* | 1.16 | 1.41 | 0.129 | 0.218 |
|  | *Oceanobacillus luteolus* | 1.76 | 0.79 | 0.484 | 0.171 |
|  | *Monoglobus pectinilyticus* | 1.27 | 1.26 | 0.214 | 0.969 |
|  | *Robinsoniella peoriensis* | 1.21 | 1.23 | 0.101 | 0.935 |
|  | *Dorea formicigenerans* | 1.22 | 1.07 | 0.146 | 0.453 |
|  | *Dorea longicatena* | 1.21 | 1.06 | 0.079 | 0.184 |
|  | *Blautia hansenii* | 1.14 | 1.04 | 0.238 | 0.761 |
| Significant results on species level | |  |  |  |  |
|  | *Roseburia hominis* | 0.48 | 0.96 | 0.077 | <0.001 |
|  | *Roseburia intestinalis* | 0.44 | 0.68 | 0.046 | 0.001 |
|  | *Roseburia faecis* | 0.29 | 0.50 | 0.041 | 0.002 |
|  | *Butyrivibrio crossotus* | 0.33 | 0.44 | 0.025 | 0.005 |
|  | *Romboutsia ilealis* | 0.24 | 0.12 | 0.039 | 0.040 |
|  | *Pseudobutyrivibrio ruminis* | 0.08 | 0.15 | 0.014 | 0.002 |
|  | *Enterococcus cecorum* | 0.02 | 0.19 | 0.020 | <0.001 |
|  | *Abiotrophia defectiva* | 0.09 | 0.04 | 0.011 | 0.007 |
|  | *Geosporobacter ferrireducens* | 0.05 | 0.08 | 0.011 | 0.048 |
|  | *Streptococcus gallolyticus* | 0.02 | 0.10 | 0.012 | <0.001 |
|  | *Lactonifactor longoviformis* | 0.03 | 0.07 | 0.010 | 0.040 |
|  | *Streptococcus pneumoniae* | 0.02 | 0.06 | 0.007 | <0.001 |
|  | *Caldicoprobacter faecalis* | 0.03 | 0.05 | 0.007 | 0.021 |
|  | *Streptococcus lutetiensis* | 0.01 | 0.06 | 0.009 | 0.007 |
|  | *Streptococcus suis* | 0.02 | 0.05 | 0.008 | 0.033 |
|  | *Anaerofustis stercorihominis* | 0.04 | 0.01 | 0.007 | 0.009 |
|  | *Marvinbryantia formatexigens* | 0.02 | 0.03 | 0.003 | 0.036 |
|  | *Streptococcus equinus* | 0.01 | 0.04 | 0.005 | 0.002 |
|  | *Terrisporobacter mayombei* | 0.03 | 0.02 | 0.004 | 0.027 |
|  | *Brevibacillus brevis* | 0.02 | 0.01 | 0.003 | 0.019 |

HMT = heat-moisture treatment; CON = control group, received feed without the HMT-modified product; HMT-Feed = treatment group, given feed containing the HMT-modified product; SEM = standard error of the mean. The data were analyzed using the Student’s t-test.


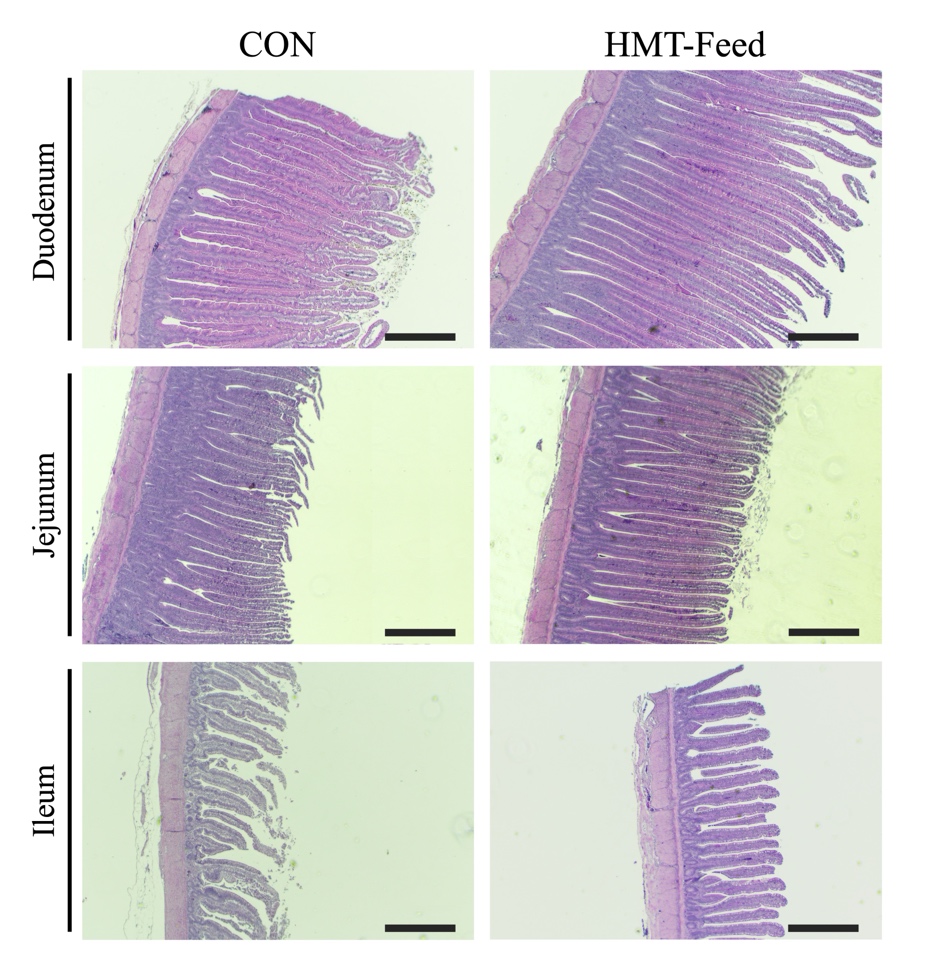


**Supplementary Fig. S1.** Histological structure of the small intestine (duodenum, jejunum and, ileum) from the CON and HMT-Feed groups, stained with H&E. Scale bar = 500 µm.
